# On the Uses of the Lobelia Inflata

**Published:** 1846-10

**Authors:** Abraham Livezey

**Affiliations:** Lumberville, Bucks county, Pa.


15. *On the Uses of the Lobelia Inflata.* By Abraham Livezey, A.M., M.D., of Lumberville, Bucks county, Pa.—Observing for several years past the use and abuse made of the lobelia by a numerous horde of quacks that abound in some parts of the country, and percieving that those dangerous consequences, which have hitherto been attributed to this plant by many of the medical profession, did not result—and that, too, when administered by a set of ignorant pretenders, in enormous doses, and almost indiscriminately in all cases—I studiously applied myself to experimental observation, to ascertain with a greater degree of certainty, the therapeutic value of this plant. And during the past year I have had many excellent opportunities of testing its beneficial influence in many diseases of febrile and spasmodic character.

In pertussis, combining the tinct. lobel., of which Professor Eberle speaks so highly, with the acid. hydrocyan., extolled by Thompson and Roe, with equal propriety might I vaunt the receipt as a specific, as they do theirs—although such a thing as a *specific* probably does not exist, except it be sulphur for psora. In asthma, especially of a spasmodic kind, the most marked benefits result from the use of this plant singly, or combined as above—the existence of the nervous fibre of the bronchial surface, or the spasms of the mucous membrane of the bronchia, are speedily allayed, and, by a short course, a cure, or a *suspension* of some length at least, is the sequence of its administration.

For an adult—℞. Tinct. lobel. inflat., ℥j.; acid. hydrocyan., gtt. i—xj. Ter quatuorve die. But if the paroxysm be severe, the tincture may be given in much larger doses, and repeated at short intervals, till entire relief is obtained. By this combination I have enabled several *inveterate* cases of asthma (which had been repeatedly prescribed for by various physicians, quacks and old women,) to pass for several months past, with a complete suspension of all their sufferings.

In diphtheritic laryngo-tracheitis, where the excitation of emesis cannot be readily accomplished, which frequently arises from the nature of the disease as well as the difficulty and unpleasantness in the administration of medicine to infants, this difficulty may be obviated by enemata containing a portion of the tinct. lobel., or pulverized plant, which at once relaxes the system, removes the tension of the chest, changes, the seat of excitement to a distant part, and emesis readily ensues; the bowels in the meanwhile are emptied of their contents, and recovery from every distressing symptom immediately follows.

In all cases of coughs, especially when inflammatory symptoms manifest themselves, as in catarrhal affections in children as well as in adults, I consider the tincture of this plant, (or infusion, when the stimulus imparted by the alcohol might be objectionable) far preferable to ipecacuanha or the tartrate of antimony and potassa, being more decisive in its effects than the former, and a better and safer nauseant than the latter, without that fear of irritating the gastro-enteric mucous membrane, the pathological condition of which has been too much overlooked by earlier writers, but which is now claiming deserved attention.

This brings me to the consideration of the lobelia inflata in febrile disorders, incident to every section of country, more or less, in summer and autumn. When it is desirable (as in fact it is always) to lessen vascular action, and as a febrifuge, the "nitrous powders" sink into utter insignificance in comparison with this plant, which is not liable to the same objection as the tartarized antimony used in combination with calomel and the nitrate of potassa by many of the older practitioners, which too frequently increases that tenderness and erethism already existing in the mucous membrane of the stomach and intestines.

In high vascular action, also, with cerebral disturbance, when the application of cups to the nape of the neck, &c., fails in restoring rationality to the sensorium, the most admirable results follow the administration of an enema, largely composed of the lobelia; or when accompanied with enervation and subsultus tendinum, the efficacy of the enema will be much enhanced by the addition of a portion of pulv. valer. and tinct. capsicum or camphor, which, when thus combined, produces a powerfully revellent action, changes the scene of excitement, and leaves the cerebral functions free.

Finally. In strangulated hernia, or in reducing dislocations of the largest articulations, where great relaxation is necessary a powerful enema of the plant, or of the bruised seeds, will fully answer the expectation of the medical attendant—attended, too, with equal benefit and much more safety than the tobacco injection used in the former difficulty, and will dispense with venesection, the tartarized antimony, and generally the hot bath, so universally recommended to overcome the rigidity of the muscular fibre.

These are the chief diseases of importance in which I have administered the lobelia inflata with entire satisfaction, and with a relief so prompt and decisive, as at once both astonished and delighted the patient.—*Medical Examiner, in Boston Med. and Surg. Journal.*

